# Hospitalized COVID-19 Patients Were Five Times More Likely to Suffer From Total Sleep Deprivation Compared to Non-COVID-19 Patients; an Observational Comparative Study

**DOI:** 10.3389/fnins.2021.680932

**Published:** 2021-10-05

**Authors:** Eva S. van den Ende, Kim D. I. van Veldhuizen, Belle Toussaint, Hanneke Merten, Peter M. van de Ven, Natasja A. Kok, Prabath W. B. Nanayakkara

**Affiliations:** ^1^Section General Internal Medicine Unit Acute Medicine, Department of Internal Medicine, Amsterdam Public Health research institute, Amsterdam University Medical Center, location VU University Medical Center, Amsterdam, Netherlands; ^2^Department of Public and Occupational Health, Amsterdam Public Health Research Institute, Amsterdam University Medical Center, Location VU University Medical Center, Amsterdam, Netherlands; ^3^Department of Epidemiology and Data Science, Amsterdam University Medical Center, Location VU University Medical Center, Amsterdam, Netherlands; ^4^Department of Pulmonary Medicine, Amsterdam University Medical Center, Location VU University Medical Center, Amsterdam, Netherlands

**Keywords:** COVID-19, Sleep quantity, sleep quality, sleep disturbing factors, hospitalized patients, comparative study, pulmonary ward

## Abstract

**Objectives:** Sleeping disorders are a common complaint in patients who suffer from an acute COVID-19 infection. Nonetheless, little is known about the severity of sleep disturbances in hospitalized COVID-19 patients, and whether these are caused by disease related symptoms, hospitalization, or the SARS-CoV-2 virus itself. Therefore, the aim of this study was to compare the quality and quantity of sleep in hospitalized patients with and without COVID-19, and to determine the main reasons for sleep disruption.

**Methods:** This was an observational comparative study conducted between October 1, 2020 and February 1, 2021 at the pulmonary ward of an academic hospital in the Netherlands. This ward contained both COVID-19-positive and -negative tested patients. The sleep quality was assessed using the PROMIS-Sleep Disturbance Short Form and sleep quantity using the Consensus Sleep Diary. Patient-reported sleep disturbing factors were summarized.

**Results:** A total of 79 COVID-19 patients (mean age 63.0, male 59.5%) and 50 non-COVID-19 patients (mean age 59.5, male 54.0%) participated in this study. A significantly larger proportion of patients with COVID-19 reported not to have slept at all (19% vs. 4% of non-COVID-19 patients, *p = 0.011*). The Sleep quality (PROMIS total score) and quantity (Total Sleep Time) did not significantly differ between both groups ((median PROMIS total score COVID-19; 26 [IQR 17-35], non-COVID-19; 23 [IQR 18-29], *p = 0.104)*, (Mean Total Sleep Time COVID-19; 5 h 5 min, non-COVID-19 mean; 5 h 32 min, *p = 0.405*)). The most frequently reported disturbing factors by COVID-19 patients were; ‘dyspnea’, ‘concerns about the disease’, ‘anxiety’ and ‘noises of other patients, medical staff and medical devices’.

**Conclusion:** This study showed that both patients with and without an acute COVID-19 infection experienced poor quality and quantity of sleep at the hospital. Although the mean scores did not significantly differ between groups, total sleep deprivation was reported five times more often by COVID-19 patients. With one in five COVID-19 patients reporting a complete absence of night sleep, poor sleep seems to be a serious problem. Sleep improving interventions should focus on physical and psychological comfort and noise reduction in the hospital environment.

## Introduction

The Severe Acute Respiratory Syndrome Coronavirus-2 (SARS-CoV-2), causing the Coronavirus Disease-19 (COVID-19) was discovered in China in December 2019 and rapidly spreaded worldwide. It was officially declared a pandemic by the World Health Organization (WHO) on the 11^th^ of March 2020 (World Health Organization [WHO], 2020). It soon became evident that besides suffering from symptoms such as dyspnea and fever, patients with COVID-19, also experienced sleeping disorders (Guo et al., 2020; Huang C. et al., 2020; Liguori et al., 2020; Mao et al., 2020; Wang et al., 2020; Yang et al., 2020).

Sleep is essential for the maintenance of a well-functioning endocrine and immune system (Cappuccio et al., 2010; Monico-Neto et al., 2020; Zhang R. et al., 2020). Even short periods of insufficient sleep or disrupted sleep-wake cycles are associated with impaired health, immune dysfunction and an induced pro-inflammation state, resulting in a higher susceptibility for (especially respiratory) infections (Irwin et al., 1996; Cohen et al., 2009; Patel et al., 2012; Prather et al., 2015; Besedovsky et al., 2019; Loef et al., 2019; Mello et al., 2020; Zhang J. et al., 2020). Sleep disturbances in COVID-19 infected patients can increase the risk of further deterioration and trigger the onset of delirium, prolong the duration of hospitalization and increase the risk of an Intensive Care Unit Admission (Huang B. et al., 2020; Zambrelli et al., 2020; Zhang J. et al., 2020). There is a bidirectional link between sleep and immunity (Besedovsky et al., 2019). Activation of the immune system during an acute infection can in turn provoke changes in the sleep-wake cycle (Besedovsky et al., 2019; Zhang R. et al., 2020).

Considering the short existence of the disease, not much is known about the sleep of hospitalized patients with an acute COVID-19 infection. A Chinese meta-analyses reported that 34% of COVID-19 patients suffered from sleep disturbances (Deng et al., 2021). But only six out of the ten studies used validated screening tools and often contained small sample sizes of Chinese out-patients. Studies which do exist, often only focus on subjective sleep quality, sleep quantity has seldom been addressed (Jiang et al., 2020). In addition, it is still unclear whether the found sleep disturbances are caused by the virus itself (e.g., due to the influence on the circadian rhythm or penetration of the virus into the cerebrospinal fluid in the central nervous system) (Besedovsky et al., 2019; Guo et al., 2020; Nalleballe et al., 2020)), an effect of symptoms (e.g., dyspnea, anxiety) (Guo et al., 2020; Deng et al., 2021) or external factors (e.g., hospitalization) (Wesselius et al., 2018).

The aim of this study was therefore to describe the quantity and quality of sleep in hospitalized patients who suffer from an acute COVID-19 infection, and to identify sleep disturbing factors. To distinguish between the direct effects of the SARS-CoV-2 virus and other factors, the sleep of COVID-19 patients was compared to that of patients admitted to the same ward due to non-COVID-19 related pulmonary problems.

## Materials and Methods

### Study Design, Participants and Eligibility

This was an observational single center comparative study conducted at the pulmonary ward of the Amsterdam University Medical Center, location VUmc, The Netherlands. The pulmonary department was split into an isolation area(patients with a positive test for SARS-CoV-2) and a regular respiratory area(for patients with a negative test for SARS-CoV-2). The study was part of a larger research project in which the sleep quantity and quality of acutely admitted hospital patients was studied. The research protocol was approved by the Medical Ethics Review Committee of VU University Medical Center Amsterdam (2019.246). Patients were recruited from the beginning of October 2020 until the end of January 2021. All patients had to be over 18 years of age, able to give written informed consent, understand the questionnaires, and admitted to the pulmonary department. Patients were excluded if they were not able to give informed consent due to cognitive impairment, severe illness or insufficient understanding of the Dutch language. Patients had to participate for at least one night, and were followed for a maximum of five consecutive nights. To minimize the patient-burden, the questionnaires of the third to the fifth nights were limited to sleep quantity assessment only. Participation ended when a patient was transferred to another ward or left the hospital. Members of the research team visited the ward seven days a week and up to three times a day to minimize the number of patients that would be missed because they were asleep or absent. Patients were asked to fill in the questionnaires as early in the morning as possible to ensure a fresh memory of the night. When needed, a member of the research team assisted with completing the questionnaire. All data was collected on paper and thereafter entered into an electronic database maintained by Castor EDC (Castor EDC, 2021) (complying with the European Data Protection Directive and ICH-GCP).

### Objectives

The aim of this study was to investigate the quality and quantity of sleep in patients hospitalized with an acute COVID-19 infection and identify reasons for the potential sleep disturbance. To distinguish between COVID-19 related and unrelated factors we compared the sleep of COVID-19 patients to that of non-COVID-19 patients at the same pulmonary ward.

The primary outcomes were the quality and quantity of sleep in patients with COVID-19, and the differences in sleep quality and quantity between COVID-19 positive and negative inpatients.

Secondary outcomes included the difference in sleep quality and quantity between COVID-19 patients with different symptoms (e.g., neurological-, pulmonary- and/or abdominal complaints), and associations between quality and quantity of sleep and patient outcomes (e.g., length of stay, admission to the Intensive Care Unit (ICU) and 30-day mortality).

### Measures

Demographic characteristics (e.g., sex, age) were recorded.

#### Sleep Quantity

The subjective sleep quantity of the preceding night was measured through self-report by the patient, for a maximum of five consecutive nights, using the Consensus Sleep Diary (CSD) (Carney et al., 2012). The CSD provided information concerning the total sleep time (TST, i.e., the actual time of being asleep), sleep onset latency (SOL, time spent awake after closing eyes to sleep), wakefulness after sleep onset (WASO, time spent awake after onset of sleep), number of awakenings (NWAK), final wake time, the time attempting to sleep after final awakening (TASAFA) and sleep efficiency (SE, the time spent asleep relative to the time trying to sleep).

#### Sleep Quality

The sleep quality as assessed by the patient was measured for a maximum of two consecutive nights. It was assessed using the 8-item Dutch-Flemish Patient-Reported Outcomes Measurement Information System (PROMIS) Sleep Disturbance Short Form (8b, version 1.0) (Terwee et al., 2014). This contained eight items concerning the experienced sleep quality of the previous night. The questions were minimally adjusted to fit the one-day assessment by changing the beginning of each question from “in the past 7 days…” into “last night…”. Each item was scored on a five-point scale. A total score for each patient was calculated, ranging from 8 to 40, with higher scores representing poorer sleep quality. From the total score, a standardized T-score was calculated. The standardized T-score facilitates comparison to a reference population (a mixture of healthy and clinical patients in the United States) which has a mean standardized T-score of 50 and a standard deviation (SD) of 10 (Hanmer et al., 2020). A patient with a T-score of 60 has a sleep quality that is worse than approximately 84% of persons in the reference population.

#### History of Insomnia

The Insomnia Severity Index (ISI) was used to quantify complaints of insomnia 30 days before admission (Morin et al., 2011). This questionnaire contained seven items, leading to a total score from 8 to 28 (0-7 = absence of insomnia; 8-14 = sub-threshold insomnia; 15-21 = moderate insomnia; 22-28 = severe insomnia).

#### Sleep Disturbing Factors

Patients were asked to give reasons in case they experienced difficulties falling asleep or staying asleep, and for waking up in the morning. Multiple choice answers that were suggested were based on sleep disturbing factors found in literature (Wesselius et al., 2018) and supplemented with open text fields.

#### Potential Confounders and Secondary Outcome Measures

Visual Analogue Scales (VAS) were used to measure depression, anxiety, pain and breathlessness (Tamiya et al., 2002). Potentially, sleep disturbances in COVID-19 patients may be explained as a neurological manifestation of the disease (Guo et al., 2020; Huang Y. H. et al., 2020; Nalleballe et al., 2020; Toscano et al., 2020). We used a checklist (e.g., headaches (yes/no), vomiting (yes/no)) to discriminate between COVID-19 patients with different symptoms (e.g., neurological-, pulmonary- and/or abdominal complaints). Literature shows an association between frailty and sleep disturbances (Wai and Yu, 2020; Balomenos et al., 2021). Therefore the Clinical Frailty Scale (CFS) was completed by the researcher after the patient consented to participate, and used to rate patients’ pre-admission frailty (Labenz et al., 2020). Scores range from 1 (very fit) to 9 (terminally ill).

Information concerning sleep disturbing or inducing medication, acute or elective admission, nocturnal vital sign checks, infusion therapy, tube feeding, timing of first medication administration, vital signs (to calculate the Modified Early Warning Score (MEWS)), Delirium Observation Scale (DOS) scores, and comorbidities [Charlson Comorbidity Index (CCI)] was collected from the medical record.

Follow-up data (i.e., length of stay, incidence of a delirium, hospital readmission, unplanned IC admission and mortality) were collected 30 days after inclusion.

### Statistical Analysis

Continuous data are summarized by their mean and standard deviation (SD) if normally distributed, and median and inter-quartile ranges (IQR) if not normally distributed. Normality was checked by visual inspection of Q-Q plots. Normal variables were compared between groups using the independent samples *t*-test, whereas variables that were not normally distributed were compared using the Mann-Whitney test. Categorical data are summarized by frequencies and percentages and compared between groups using chi-square test or Fisher’s exact test in case of (expected) cell counts under five. To investigate associations between sleep quality, sleep quantity, experienced emotions and symptoms (i.e., fear, depression, pain, dyspnea) Spearman correlations were calculated. To adjust for potential confounding (i.e., by age, gender, number of patients in the room, dexamethasone use and VAS-scores) linear regression analysis was used. The main analyses were conducted separately for all consecutive days of participation and are shown in the Supplementary Material. In the manuscript, the focus was put on the results of the first night due to the large decrease in sample size with each consecutive day and to ensure readability and clarity of the results. P-values of <0.05 were considered statistically significant. All analysis were performed using Statistical Package for Social Sciences for Windows (SPSS), version 26.

## Results

### Patient Characteristics

**FIGURE 1:**
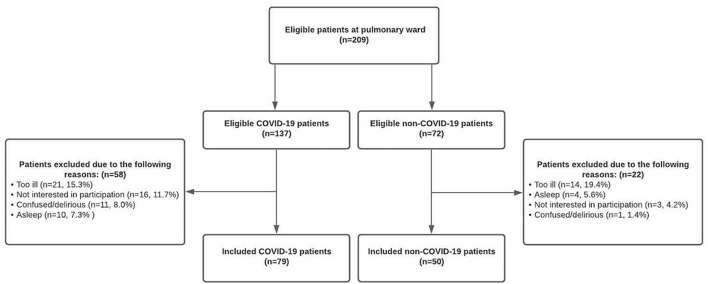
Flowchart of patient inclusion and exclusion.

A total of 209 patients were potentially eligible for inclusion in this study. However, some patients were too ill to participate (COVID-19; 15.3% [21/137] non-COVID-19; 19.4% [14/72]), others were not interested (COVID-19; 11.7% [16/137] non-COVID-19; 4.2% [3/72]), too confused (COVID-19; 8.0% [11/137] non-COVID-19; 1.4% [1/72]) or continuously asleep (COVID-19; 7.3% [10/137] non-COVID-19; 5.6% [4/72]) at the day of inclusion. Therefore, 129 patients (61.7%) were successfully included, of whom 79 (61.2%) suffered from an acute COVID-19 infection and 50 patients (38.8%) were admitted due to non-COVID-19 related pulmonary complaints (Figure 1).

In both groups, just over half were male (COVID-19; 47 [59.5%] non-COVID-19; 27 [54.0%]). The median age of the COVID-19 group was slightly higher than in the non-COVID-19 group (63.0 [IQR, 56.0-73.0] years and 59.5 [IQR, 50-68] years respectively). Nevertheless, the non-COVID-19 group scored a higher median [IQR] CCI-score (4 [2-6.5] vs. 3 [1.5-4.0]) indicating more life-threatening comorbidities. More patients in the COVID-19 group received oxygen therapy (COVID-19; 58 patients (73.4%); non-COVID-19; 12 patients (24.0%)). The median number of liters oxygen/minute was 3.0 liters [2.0-5.0] for COVID-19 patients and 2.0 liters [1.3-2.8] for non-COVID-19 patients. Benzodiazepines were used by 14.3% (*n* = 11) of the COVID-19 patients versus 18% (*n* = 9) of the non-COVID-19 patients. Opioids were more often used in the non-COVID-19 group (28.0% (*n* = 14) vs. 5.1% (*n* = 4)), Dexamethasone was more administered to COVID-19 patients (COVID-19; 64.9% (*n* = 50), non-COVID-19; 8% (*n* = 4)). There were no notable differences in the administration of other potentially sleep affecting medications (Supplementary Table 1). For a majority of patients in both groups, the first night in the study was their first or second night in the hospital. No considerable differences were found in length of stay, history of insomnia, CFS, MEWS and DOS scores. Patient characteristics are presented in Table 1. Supplementary Table 2 shows the patient status 30 days after inclusion.

**TABLE 1:** Baseline characteristics of patients with and without COVID-19.

Characteristic	COVID-19	Non-COVID-19
	(*n* = 79)	(*n* = 50)
Gender, male, n (%)	47 (59.5)	27 (54.0)
Age, median [IQR]	63.0 [56.0–73.0]	59.5 [50.0–68.0]
**Age-groups**		
<50	8 (10.4)	12 (24.5)
50–59	22 (28.6)	13 (26.5)
60–69	20 (26.0)	14 (28.6)
70–79	18 (23.4)	10 (20.4)
≥80	9 (11.7)	0 (0)
**Tested on COVID-19 by PCR, n (%)**		
Positive result	77 (100)	2 (4.0)^a^
Negative result	0 (0.0)	31 (62.0)
Not tested	0 (0.0)	17 (34.0)
Length of Stay, median [IQR]	5.50 [4.00–9.25]	5.00 [4.00–10.00]
No. of nights hospitalized at first night of participation, median [IQR]	2.0 [1.0–3.0]	2.0 [1.0–3.0]
**No. of nights participated in study, n (%)**		
1 night	79 (100)	50 (100)
≥*2* nights	55 (69.9)	37 (74.0)
Other patients in the room, median [IQR]	2 [1–3]	1 [0.25–2.0]
**Other patients in the room, n (%)**		
0	13 (16.7)	12 (25.0)
1	9 (11.5)	15 (31.3)
2	28 (35.9)	17 (35.4)
3	28 (35.9)	4 (8.3)
**Location of patient before inclusion, n (%)**		
Home/Nursing Home/Rehabilitation Centre	51 (64.6)	38 (76.0)
Intensive Care Unit	8 (10.1)	3 (6.0)
Other ward/hospital (not ED)	19 (24.1)	9 (18.0)
Missing	1 (1.3)	0
**History of Insomnia^b^, n (%)**		
Absence of insomnia	52 (68.4)	35 (74.5)
Sub-threshold insomnia	11 (14.5)	7 (14.9)
Moderate insomnia	10 (13.2)	3 (6.4)
Severe insomnia	3 (3.9)	2 (4.3)
Clinical Frailty Scale^c^, median [IQR]	3 [2–4]	3 [2–3]
Charlson Comorbidity Index^d^, median [IQR]	3 [1.5–4.0]	4 [2–6.5]
Modified Early Warning Score^e^, median [IQR]	1 [0–1]	0 [0–1]
No. of patients that received oxygen therapy at day 1, n (%)	58 (73.4)	12 (24.0)
Missing	2 (2.5)	1 (2.0)
Liters oxygen/minute, median [IQR]	3.0 [2.0–5.0]	2.0 [1.3–2.8]
Delirium Observation Scale^f^, median [IQR]	0 [0.00–1.75]	0 [0.00–0.00]
Missing, n (%)	71 (89.9)	49 (98.0)

*COVID, coronavirus disease; IQR, interquartile range; ED, Emergency Department.*

*^*a*^Two patients had tested positive for COVID-19 in an earlier phase of their admission.*

*^*b*^Measured by means of the Insomnia Severity Index (ISI), resulting in a total score ranging from 0 to 28. Absence of insomnia (0–7), sub-treshold insomnia (8–14), moderate insomnia (15–21) and severe insomnia (22–28).*

*^*c*^The Clinical Frailty Scale (CFS) is used to rate patients’ pre-admission frailty. Scores range from 1 (very fit) to 9 (terminally ill).*

*^*d*^The Charlson Comorbidity Index (CCI) results in a total score ranging from 0 to 24. No comorbidity (0), mild comorbidity (1–2), moderate comorbidity (3-4), severe comorbidity (≥5).*

*^*e*^The Modified Early Warning Score (MEWS) is mainly based on vital parameters (i.e., systolic blood pressure, heart rate, respiratory rate, temperature, AVPU score) and used for identification of patients at risk of deterioration. Each parameter is scored from 0 to 3, resulting in a total score ranging from 0 (best) to 14 (worst).*

*^*f*^The Delirium Observation Scale (DOS) is designed to identify symptoms of delirium. A total score of 3 or more suggests delirium. DOS scores were subtracted from the electronic health record. Nurses only take DOS scores if suspecting a delirium explaining the large number of missing values.*

### Sleep Quantity

Of all patients in the COVID-19 group, 19% (13/70) reported to not have slept at all, versus 4% (2/45) of patients in the non-COVID-19 group (*p = 0.011)*. Within the COVID-19 group, patients who reported a total absence of night-sleep where significantly older (0 h sleep; median age 73.0 years [IQR 63.0-83.0], ≥ 1 h of sleep; 62.0 years [IQR 53.8-72.0] (*p = 0.010*)) and were slightly more frail (0 h sleep; median CFS 4 [IQR 3-5], ≥ 1 h of sleep; 3 [IQR 2-4] (*p = 0.037*)) than patients that did get some sleep at night. There were no statistical differences between COVID-19 patients with and without sleep in terms of other basic characteristics (e.g., gender, number of patients in the room, history of insomnia), symptoms (e.g., VAS-scores for depression, dyspnea) or patient outcomes (e.g., 30-day mortality) (Supplementary Table 3).

**FIGURE 2:**
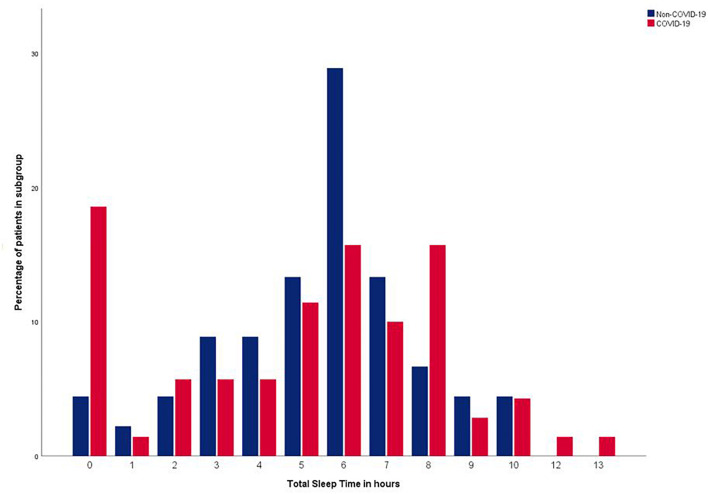
Distribution of Total Sleep Time within COVID-19 and non-COVID-19 group.

Figure 2 shows the distribution of the Total Sleep Time of patients with and without COVID-19, which is a mixture of a symmetric distribution centered around 6 h (range 0-10 h) and a small peak at 0 h for the non-COVID-19 group and a wider distribution centered around 6 h (range 0-13 h) with a larger peak at 0 h for the COVID-19 group. The mean Total Sleep Time was 26 min shorter in the COVID-19 group (05 h 05 min [SD 03:18]) compared to the non-COVID-19 group (05 h 32 min [SD 02:17]) the first night of participation, yet no significant p-value was found (*p = 0.405)*. Neither was a statistical significant difference found when comparing the median Total Sleep Times (COVID-19; 05 h 43 min [IQR hh:mm: 02:24-07:40], non-COVID-19 06 h 00 min [IQR hh:mm 04:15-06:50] (*p = 0.618*)). In three out of the four consecutive nights, the same pattern was found; a shorter Total Sleep Time in the COVID-19 group, without a statistically significant difference (see Table 2 and Supplementary Table 4).

**TABLE 2:** Sleep quantity by means of the Consensus Sleep Diary.

	Day 1				Day 2			
		
	COVID-19	Non-COVID-19	Difference	*P*-value	COVID-19	Non-COVID-19	Difference	*P*-value
	(*n* = 79)	(*n* = 50)			(*n* = 55)	(*n* = 36)		
**Closing Eyes to Sleep Time**								
Mean (SD)	22:57 (1:48)^c^	23:16 (1:19)^a^	−00:19	0.301	22:52 (01:38)	23:15 (01:22)	−00:23	0.258
Median [IQR]	23:00 [22:00–00:00]^c^	23:15 [22:45–23:52]^a^	−00:15	0.083	23:00 [22:00–24:00]	23:00 [22:30–23:55]	0:00	0.14
**Sleep Onset Latency^1^**								
Mean (SD)	00:58 (01:33)^j^	00:43 (00:53)^e^	0:15	0.265	00:42 (00:58)^a^	00:30 (00:44)^a^	0:12	0.321
Median [IQR]	00:15 [00:10–01:00]^j^	00:30 [00:10–00:53]^e^	−00:15	0.611	00:25 [00:06–01:00]^a^	00:15 [00:05–00:30]^a^	0:10	0.2
**Number of Awakenings, No.**								
Mean (SD)	3.6 (4.6)^g^	3.2 (2.4)	0.4	0.533	2.4 (2.4)^f^	2.6 (3.5)	−0.2	0.769
Median [IQR]	2.0 [1.0–4.0]^g^	3.0 [1.0–5.0]	−1	0.477	2.0 [1.0–3.0]^f^	2.0 [1.0–3.0]	0	0.935
**Wake After Sleep Onset^2^**								
Mean (SD)	01:07 (01:34)	00:55 (00:59)^b^	0:12	0.438	00:50 (01:02)^h^	01:04 (01:09)^d^	−00:14	0.343
Median [IQR]	00:30 [00:10–01:30]	00:40 [00:10–01:23]^b^	−00:10	0.9	00:30 [00:05–01:00]^h^	00:43 [00:05–01:30]^d^	−00:13	0.301
**Final Wake Time**								
Mean (SD)	06:24 (01:34)^e^	06:29 (00:47)^a^	−00:05	0.691	06:11 (01:36)	06:15 (00:50)	−00:04	0.799
Median [IQR]	06:20 [05:30–07:00]^*e*^	06:20 [06:00–07:00]^a^	0:00	0.919	06:00 [05:30–07:00]	06:00 [05:37–06:30]	0:00	0.949
**Sleep Episode^3^**								
Mean (SD)	06:42 (03:18)	06:59 (02:05)07:13 [06:00–08:00]	−00:17	0.54	07:17 (01:53)^b^	06:55 (01:35)^a^	0:22	0.338
Median [IQR]	07:15 [06:00–08:30]		0:02	0.985	07:00 [06:08–08:28]^b^	07:00 [05:30–08:00]^a^	0:00	0.405
**Total Sleep Time^4^**								
Mean (SD)	05:05 (03:18)^i^	05:32 (02:17)^e^	−00:26	0.405	05:22 (02:49)^h^	05:25 (02:21)^e^	−00:03	0.933
Median [IQR]	05:43 [02:24–07:40]^i^	06:00 [04:15–06:50]^e^	−00:17	0.618	05:55 [03:45–07:40]^h^	05:45 [04:15–07:00^e^	0:10	0.898
**Sleep Efficiency^5^, %**								
Mean (SD)	75.9 (26.9)	78.5 (19.5)^e^	−2.6	0.58	71.0 (31.5)^h^	74.7 (27.8)^e^	−3.7	0.598
Median [IQR]	82.64 [31.41–93.01]^g^	82.35 [64.58–94.91]^e^	0.29	0.283	84.95 [60.00–93.59]^h^	87.04 [57.58–95.45]^e^	−2.09	0.721
**Time Attempting to Sleep After Final Awakening**								
Mean (SD)	00:53 (01:10)^h^	00:30 (00:49)^d^	0:23	0.042	00:56 (01:25)^f^	00:23 (00:32)^a^	0:33	0.017
Median [IQR]	00:30 [00:00–01:30]^h^	00:00 [00:00–00:49]^d^	0:30	0.111	00:15 [00:00–01:30]^f^	00:05 [00:00–01:00]^a^	0:10	0.12
**Daytime Sleep**								
Mean (SD)	00:59 (01:36)	00:46 (01:55)	0:13	0.511	00:45 (01:08)	00:40 (01:17)	0:05	0.797
Median [IQR]	00:00 [00:00–01:37]	00:00 [00:00−01:00]	0:00	0.104	00:00 [00:00 01:00]	00:00 [00:00−01:00]	0:00	0.431

*IQR, interquartile range; min, minutes; No., number. All data are presented as hours:minutes unless indicated otherwise. Difference indicate COVID-19 minus non-COVID-19 scores. Questions containing missing values;^*a*^one missing, ^*b*^two missing, ^*c*^three missing, ^*d*^four missing, ^*e*^five missing, ^*f*^six missing, ^*g*^seven missing, ^*h*^eight missing, ^*i*^nine missing, ^*j*^ten missing.*

*^1^Time it took to fall asleep after closing eyes to sleep.*

*^2^Time spent awake after onset of sleep.*

*^3^Time interval from “Closing eyes to sleep” until “final awakening”.*

*^4^The time spent asleep within the sleep episode.*

*^5^The percentage of sleep time within the sleep episode (TST/SE (^∗^100)).*

Separate questions of the Consensus Sleep Diary (i.e., Sleep Onset Latency, Number of Awakenings, Time Spent Awake after Onset of Sleep, Final Wake Time and Sleep Efficiency) were all answered more negatively in the COVID-19 group than in the non-COVID-19 group, but no significant differences were found. Time Attempting to Sleep After Final Awakening was significantly longer for the COVID-19 (52.6 min [SD 70.3]) than for the non-COVID-19 group (30.0 min [SD 48.5]) (*p = 0.042*) (see Table 2).

No clear improvement or deterioration of the sleep quantity over the consecutive days was found within the individual patients who participated for five days in both sub-groups (Supplementary Figure 1).

Of all potential confounders (age, gender, dexamethasone use, number of other patients in the room and VAS-scores) only age and dexamethasone use showed a more than 10% change in the regression coefficient when performing linear regression analyses. However, no significant difference was found between the COVID-19 and non-COVID-19 group in terms of mean Total Sleep Time after correction for these confounders.

### Sleep Quality

No significant difference was found in the PROMIS total score for the first night of participation (COVID-19; median 26 [IQR 17-35], non-COVID-19; median 23 [IQR 18-29], *p = 0.104*) nor for the second night of participation (COVID-19; median 23 [IQR 14.5-33.0], non-COVID-19; median 22 [IQR 14-29], *p = 0.336*). On average, COVID-19 patients scored the same or higher on seven out of eight PROMIS-items indicating an equal or poorer sleep-quality than that of patients in the non-COVID-19 group (although the difference was only statistically significant for overall sleep satisfaction and for experienced trouble getting enough sleep). A full representation of the PROMIS items can be found in Table 3.

**TABLE 3:** Sleep quality measured by means of PROMIS Sleep Disturbance Scores.

	Day 1				Day 2			
		
	COVID-19 (*n* = 79)	Non-COVID-19 (*n* = 50)	Difference	p-value	COVID-19 (*n* = 55)	Non-COVID-19 (*n* = 36)	Difference	p-value
My sleep was restless (not at all (1) - very much (5))	3 [2-5]	3 [2-4]	0	0.059	2 [1-4]	2.5 [1-4]	−0.5	0.690
I was satisfied with my sleep (very much (1) - not at all (5))	4 [2-5]	3 [2-4]	1	0.012	3 [2-5]	3 [2-4]	0	0.322
My sleep was refreshing (very much (1) - not at all (5))	4 [2-5]	3 [2-4]	1	0.211	4 [2-5]	3 [2-4]	1	0.374
I had difficulty falling asleep (not at all (1) - very much (5))	2 [1-4]^a^	2.5 [1-4]	−0.5	0.485	2 [1-4]^a^	2 [1-3]	0	0.817
I had trouble staying asleep (not at all (1) - very much (5))	4 [2-5]	3 [2-4]	1	0.350	3 [2-5]	3 [2-4]^a^	0	0.436
I had trouble sleeping (not at all (1) - very much (5))	3 [1-5]	3 [1-4]	0	0.169	2 [1-4]	2 [1-4]	0	0.537
I got enough sleep (very much (1) - not at all (5))	4 [2-5]	3 [2-4]	1	0.030	3 [2-5]	3 [2-4]	0	0.590
My sleep quality was (very good (1) - very poor (5))	3 [2-4]	3 [2-4]	0	0.106	3 [2-4]^a^	3 [2-4]	0	0.559
Raw PROMIS total score	26 [17.0-35.3]^a^	23 [18.0-28.8]	3.0	0.104	23 [14.5-33.0]^b^	22 [14.0-29.0]	1.0	0.366
Standardized score^1^, T-score (SE)	56.3 (2.5)	53.3 (2.5)			53.3 (2.5)	52.2 (2.5)		

*IQR, interquartile range; PROMIS, Patient-Reported Outcomes Measurement Information System.*

*Data are presented as median [IQR] unless indicated otherwise.*

*The PROMIS-Sleep disturbance 8b (Short Form) was used. Every question was answered using a 5-point scale, a higher score representing a more negative sleep-experience. The total score ranges from 8-40, a higher meaning more sleep disturbance.*

*^1^The T-score is a standardized score based on a mixture of healthy and clinical patients in the United States and calculated from the Raw PROMIS total score. The T-score has a mean of 50 and a standard deviation (SD) of 10. A person with a T-score of 60 is one SD worse than the reference population.*

*^*a*^For this question there was one missing value.*

*^*b*^The Raw PROMIS total score for day 2 could not be calculated for 2 patients due to missing values.*

### Sleep-Disturbing Factors

Factors causing difficulties falling asleep were in COVID-19 patients mostly (59.7%) intrinsic (i.e., patient and illness related) and in non-COVID-19 patients extrinsic (64.1%; i.e., environmental factors). The same pattern was found for factors causing nocturnal awakenings (COVID-19; 53.7% intrinsic factors, non-COVID-19; 56.4% extrinsic factors). The final awakening in both groups was mostly caused by extrinsic factors (COVID-19; 68.8%, non-COVID-19; 69.1%), see Table 4.

**TABLE 4:** Sleep-Disturbing Factors COVID-19 and non-COVID-19 patients during the first night.

		Prolonged Sleep Onset		Nocturnal Awakenings		Final Awakening	
				
		COVID-19	Non-COVID-19	COVID-19	Non-COVID-19	COVID-19	Non-COVID-19
**Extrinsic vs. Intrinsic factors^a^**							
		40.3%/59.7%	64.1%/35.9%	46.3%/53.7%	56.4%/43.6%	68.8%/31.2%	69.1%/30.9%
**Extrinsic Factors Top 3^b^**							
	1	Noises of other patients (21.5 %)	Noises of other patients (28.0%)	Noises of other patients (25.3%)	Noises of other patients (32.0%)	Awakened by hospital staff (61.2%)	Awakened by hospital staff (54.0%)
	2	Noises from medical devices (13.9%)	Noises of hospital staff (22.0%)	Awakened by hospital staff (20.3%)	Noises of hospital staff (24.0%)	Noises of hospital staff (7.6%)	Noises of hospital staff (12.0%)
	3	Lights (8.9%)	Noises from medical devices/Awakened by hospital staff (16.0%)	Noises from medical devices (12.7%)	Noises from medical devices (18.0%)	Noises of other patients/Lights (5.1%)	Noises from medical devices (8.0%)
**Intrinsic Factors Top 3^b^**							
	1	Dyspnea **/** Concerns about illness (19.0%)	Concerns about illness (20.0%)	Toilet visit (24.1%)	Toilet visit (34.0%)	Spontaneous (22.8%)	Spontaneous (22.0%)
	2	Anxiety (15.8%)	Pain/Too hot (12.0%)	Dyspnea (15.8%)	Pain (16.0%)	Toilet visit (6.3%)	Toilet visit/Self-set alarm (4.0%)
	3	Other intrinsic factors^c^ (17.7%)	Dyspnea **/** Anxiety (8.0%)	Concerns about illness (13.9%)	Too hot (12.0%)	Other intrinsic factors^c^ (6.0%)	Pain/Dyspnea **/** Anxiety **/** Concerns about illness/Too hot/cold (2.0%)

*Table includes data from day 1 (COVID-19 n = 79, Non-COVID-19 *n* = 50).*

*Patients were allowed to assign multiple sleep disturbing factors to why they experienced a prolonged sleep onset latency and/or nocturnal awakenings. Only one answer could be assigned to what caused their final awakening.*

*^*a*^Percentages are the proportion of all sleep-disturbing factors mentioned for that moment in the night within that sub-group. Patients were allowed to assign multiple sleep disturbing factors to why they experienced a prolonged sleep onset latency and/or nocturnal awakenings. Only one answer could be assigned to what caused their final awakening.*

*^*b*^Top 3 most mentioned disturbing factors. Percentage of all patients in that sub-group (COVID-19/Non-COVID-19) that suffered from that sleep-disturbing factor.*

*^*c*^Other intrinsic factors e.g., general complaints due to COVID-19 infection, symptoms (i.e., coughing, headaches, dizziness, gastro-intestinal reflux, sweating).*

The main extrinsic factors causing difficulties falling asleep were for both COVID-19 and non-COVID-19 patients the same; ‘noises of other patients’, ‘noises of hospital staff’ and ‘noises of medical devices’. The intrinsic reasons for not falling asleep that were most reported by COVID-19 patients were ‘dyspnea’ reported by 19.0% of COVID-19 patients, 8.0% of non-COVID-19 patients, and ‘concerns about the illness’ (COVID-19; 19.0%, non-COVID-19; 20.0%).

The main extrinsic reasons for nocturnal awakenings were again ‘noises of other patients’, ‘noises of hospital staff’ and ‘noises of medical devices’ for both groups. COVID-19 patients reported more often than non-COVID-19 patients to be awakened by hospital staff during the night (20.3% vs. 14.0%, Supplementary Figures 2a-c). Intrinsic reasons were in both groups ‘the need for toilet visits’ (24.1% vs. 34.0%), for COVID-19 patients ‘dyspnea’ (15.8%) and ‘concerns about the illness’ (13.9%), and for non-COVID-19 patients ‘pain’ (16.0%) and ‘feeling too hot’ (12.0%).

Most patients reported to be finally awakened by hospital staff (COVID-19; 61.2%, non-COVID-19; 54.0%) or the noises of hospital staff (COVID-19; 7.6%, non-COVID-19; 12.0%).

Table 4 shows the top 3 sleep-disturbing factors for COVID-19 and non-COVID-19 patients during the first night. Results differ slightly between the first and second night. A full overview of all extrinsic and intrinsic factors for both nights can be found in Supplementary Figures 2a-c.

### Depression, Anxiety, Pain and Dyspnea

Patients in the COVID-19 group scored significantly higher (worse) on the depression and anxiety VAS-scale than patients in the non-COVID-19 group (depression; median 4 [IQR 1-7], median 1.5 [IQR 1-6] respectively (*p = 0.016)*. Anxiety; median 4 [IQR 1-7], median 2 [IQR 1-4] respectively (*p = 0.015)*). The same pattern was found for dyspnea, both in rest and while walking to the toilet, with higher (worse) median scores for COVID-19 patients and p-values for difference scores approaching significance (median score in rest COVID-19; 3 [IQR 1-6], non-COVID-19; 2 [IQR 1-4] (*p = 0.052)*. Median score while walking COVID-19; 6 [IQR 2-9], non-COVID-19; 4 [IQR 2-6] (*p = 0.052)*). The second day questionnaire revealed the same trends (higher scores for depression, anxiety and dyspnea in the COVID-19 group) but only the difference score for pain showed statistical significance (COVID-19 median 1 [IQR 1-2.25], non-COVID-19 median 4 [IQR 2-5] (*p = 0.000))* (Table 5).

**TABLE 5:** Visual Analog Scale (VAS) for depression, anxiety, pain and dyspnea.

	Day 1				Day 2			
		
	COVID-19 (*n* = 79)	Non-COVID-19 (*n* = 50)	Difference	P-value	COVID-19 (*n* = 55)	Non-COVID-19 (*n* = 36)	Difference	P-value
Depression	4 [1-7]^a^	1.5 [1-6]	2.5	0.016	4 [1-7]^b^	2 [1-5]^a^	2	0.341
Anxiety	4 [1-7]^b^	2 [1-4]	2	0.015	2 [1-7.5]^b^	1 [1-4]^a^	1	0.110
Pain	1 [1-4]^c^	2 [1-5]	1	0.171	1 [1-2.25]^a^	4 [2-5]^*a*^	3	0.000
Dyspnea in rest	3 [1-6]^c^	2 [1-4]	1	0.052	3 [1.4.25]^a^	2 [1-4]^a^	1	0.074
Dyspnea while walking^1^	6 [2-9]	4 [2-6]	2	0.052	5 [2-7]	2.5 [1-5]	2.5	0.063

*Data are presented as median [IQR].*

*All VAS scores result in a total score ranging from 0-10, whereat 0 represents not being depressed, no anxiety, no pain and no shortness of breath. The maximum score of 10 stands for feeling depressed, being very anxious, feeling the worst possible pain and being maximally short of breath.*

*^*a*^This question contains one missing value.*

*^*b*^This question contains two missing values.*

*^*c*^This question contains three missing values.*

*^1^Patients were asked to rate their level of dyspnea when walking to the toilet. Patients who could not walk were not able to answer the question. Therefore, there were sixteen missing values in the COVID-19 group, and eight missing values in the non-COVID-19 group on day 1. For day 2 this was six in both groups.*

### Correlations of Sleep, Experienced Symptoms and Patient-Outcomes

COVID-19 patients with different types of complaints (i.e., pulmonary, neurological, abdominal or muscle/joint complaints) did not experience significant differences in sleep quality (PROMIS total score) or quantity (Total Sleep Time). See Supplementary Table 5.

Within the COVID-19 group, the subjective sleep quality (PROMIS total score) was found to be associated with anxiety (r 0.27, *p = 0.017)* and depression (r 0.303, *p = 0.007*), but not with pain or dyspnea. There were no significant correlations between the PROMIS total score and patient outcomes (i.e., length of stay, unplanned ICU admission, thirty-day mortality).

The sleep quantity (Total Sleep Time) was neither correlated with depression, anxiety, pain or dyspnea, nor with unplanned ICU admissions or thirty-day mortality. Analysis did show a low positive correlation with length of stay (r 0.303, *p = 0.011*). Sleep quality (PROMIS total score) and quantity (Total Sleep Time) were strongly correlated (r -0.542, *p ≤ 0.001*). All correlation coefficients are shown in Supplementary Table 6.

Lastly, an increase in patient reported physical well-being (compared to the day before) was correlated with a patient reported increase in the experienced sleep quality (compared to the night before) (r 0.300 *p = 0.005*) (Supplementary Figure 3).

## Discussion

Not much was known about the severity of sleep disturbances in patients hospitalized with an acute COVID-19 infection and whether these sleep disturbances were caused by the SARS-CoV-2 virus itself or other factors (Jiang et al., 2020; Vitale et al., 2020). By including patients on the same ward with similar complaints but without an acute COVID-19 infection as a comparison, we were able to fill some of these knowledge gaps.

There were no statistically significant differences between the two groups in terms of self-assessed sleep quality (PROMIS total score) and quantity (Total Sleep Time). With standardized PROMIS T-scores above 50, both patients with and without COVID-19 suffered from worse sleep quality than the PROMIS reference population of healthy and clinical patients in the United States (Hanmer et al., 2020; The Interpretation of Promis Scores, 2021). The mean sleep quantity in both groups (5 h 5 min in the COVID-19 group, and 5 h 32 min in the non-COVID-19 group (*p = 0.405*)), was on all five days less than the minimum of 7 h sleep for adults as recommended by the National Sleep Foundation (Watson et al., 2015; Ohayon et al., 2017; Pelayo, 2017). It was also less than found in the general Dutch hospital population (6 h and 4 min) by Wesselius et al. (2018) and the mean sleep duration of 6 h 3 min that was measured in a case series of four inpatients recovering from COVID-19 (Vitale et al., 2020).

Although we found the mean Total Sleep Time in patients with and without COVID-19 to be roughly the same, the data showed a clear difference in the distribution of Total Sleep Time between both groups. In the non-COVID-19 group it followed an approximately normal distribution around 6 h of sleep (with a small number of patients not sleeping at all, or up to 10 h), whereas the COVID-19 group showed a high percentage of patients who suffered from either total sleep deprivation, or excessive sleep duration (up to 13 h). Nearly one in five patients in the COVID-19 group reported not to have slept at all, versus one in twenty-five patients in the non-COVID-19 group. Both undersleeping and oversleeping are associated with adverse health outcomes and even mortality, underscoring the clinical relevance of this finding (Cappuccio et al., 2010; Watson et al., 2015).

Literature shows that during an acute infection, sleeping hours can either be reduced or prolonged due to the activation of the immune system, offering a plausible explanation for the wide distribution of Total Sleep Time in COVID-19 patients (Besedovsky et al., 2019; Nalleballe et al., 2020; Vitale et al., 2020). The association between sleep and functioning of the immune system is bidirectional. Night-sleep is regulated by a circadian rhythm that is strongly dependent on the release of the hormone melatonin (Dubocovich, 2007). Since melatonin has anti-inflammatory and immunomodulatory effects, a decrease in night-time melatonin levels is thought to increase the susceptibility for the SARS-CoV-2 virus. Melatonin has even been suggested as an adjunctive therapy in patients with COVID-19 (Ramos et al., 2021). The secretion of melatonin is synchronized with the rhythm of the immune system. Therefore, a disrupted circadian rhythm will increase the risk of infection. Poor sleep leads to an increase in receptors for pro-inflammatory cytokines such as IL-6 and TNF-alpha, and decrease of receptors for anti-inflammatory cytokines such as IL-10 (Lange et al., 2010). In turn, cytokines are known to disrupt both non-REM and REM sleep (Majde and Krueger, 2005), accounting for poor sleep at night, daytime sleepiness and an increased need to nap (Lentz et al., 1999; Besedovsky et al., 2019). COVID-19 is often associated with high levels of pro-inflammatory cytokines (Henderson et al., 2020).

Poor night-sleep as a result of changes in the circadian rhythm can also be explained by the hospital stay itself. The circadian rhythm is mainly triggered by daylight, timing of meals and exercise (Tahara and Shibata, 2018). These three factors are often poorly available, or different from home during a hospital stay.

Sleeping disorders, encephalopathy, and mood disorders are in some cases considered as neurological manifestations of COVID-19 (Guo et al., 2020; Huang Y. H. et al., 2020; Nalleballe et al., 2020; Toscano et al., 2020). Neurological complaints are being attributed to the SARS-CoV-2 virus invading the central nervous system, hypoxic brain injury due to low oxygen levels or stroke, activated microglia attacking neurons, or inflammation of the brain’s blood vessels (Duarte-Neto et al., 2020; Reichard et al., 2020; Solomon et al., 2020; Thakur et al., 2021). This study showed no significant differences in sleep quality nor quantity between COVID-19 patients with and without neurological complaints (i.e., headaches, memory loss).

In accordance with the earlier research, extrinsic (environmental factors) as well as intrinsic factors (physiological and psychological discomfort) were subjectively responsible for sleep disturbance in both groups (Wesselius et al., 2018; Zhang J. et al., 2020). However, patients with an acute COVID-19 infection complained more often of intrinsic factors than non-COVID-19 patients, suggesting a greater contribution of the disease in terms of sleep disturbance. This could be explained by a higher burden of the disease, or less distraction from the visitors and the staff due to the isolation measures. The main intrinsic factors keeping these patients from sleeping were ‘dyspnea’, ‘concerns about the disease’ and ‘anxiety’. Dyspnea, symptoms of depression, anxiety and mental distress are often found in patients with COVID-19 (Casagrande et al., 2020; da Silva et al., 2020; Guo et al., 2020; Kong et al., 2020; Wu and McGoogan, 2020), but could, of course, also be common in patients with non-COVID-19 related pulmonary-problems. Nevertheless, our study showed that patients in the COVID-19 group scored significantly higher (worse) on the Visual Analog Scales for depression, anxiety and dyspnea than patients in the non-COVID-19 group. The same was found by Guo et al. after comparing the depression and anxiety-levels between COVID-19 patients and non-COVID-19 controls (Guo et al., 2020). The higher burden of these symptoms could therefore be a potential reason for COVID-19 patients to sleep worse than the comparison group. Psychological distress is known to impair sleep by activation of the hypothalamic-pituitary-adrenal (HPA) axis, and in turn, poor sleep increases the HPA activation leading to a vicious cycle of distress and poor sleep (Akerstedt, 2006). In this study, a higher anxiety-rate did indeed negatively correlate with the overall experienced sleep quality. Nevertheless, none of these symptoms was negatively associated with sleep quantity, possibly this relation follows a rather non-linear inverted-U shaped curve. Earlier research shows that sleep quality is indeed better related to health, stress, depression, anger and tiredness than sleep quantity (Pilcher et al., 1997).

Although no clear correlations between sleep quality and quantity and patient outcomes (length of stay, unplanned ICU admission, thirty-day mortality) were found in this study, literature shows strong correlations between disturbed sleep in (COVID-19) patients and prognosis (Bijwadia and Ejaz, 2009; Friese et al., 2009; Zhang J. et al., 2020). A retrospective cohort study showed that sleep quality in hospitalized patients with COVID-19 was associated with an impaired recovery from lymphopenia and increased chances of ICU admissions (Zhang J. et al., 2020). Even short-term sleep disturbances are known to generate a drop in natural killer cell activity, an increase in upper respiratory infections and higher chances of mortality (Irwin et al., 1996).

This study shows that patients with COVID-19 are prone to suffer from seriously disrupted night-sleep. Knowing the negative effects on psychological and physical well-being and prognosis, optimizing sleep should play an important role in the treatment of this infection. Interventions should focus on environmental factors on the ward (e.g., reducing noise of hospital staff, other patients and medical devices were possible, and maintaining a healthy circadian rhythm), psychological treatment (e.g., emotional support) and symptom relief (e.g., alleviating breathing difficulties, adequate medication). Given the possible beneficial effects of melatonin during a COVID-19 infection and the minor side effects if used correctly, administration of melatonin could be considered (Corrao et al., 2021; Ramos et al., 2021).

Several limitations of the current study need to be raised. Firstly, due to the risk of transmission of the infection, it was not possible to use polysomnography (as is deemed the gold standard for measuring sleep objectively (Van de Water et al., 2011)) or any other measuring device. Therefore, sleep quantity was measured by means of the Consensus Sleep Diary, a standardized questionnaire that is often used in sleep-research (Maich et al., 2018). Secondly, although patients were followed for up to five consecutive days, this paper focusses mainly on the data collected on day one of participation, because of the large drop in sample size with each consecutive day and to increase the readability and interpretation of the results. Results for all days can be found in the Supplementary Material. Analyses showed no notable differences in sleep between consecutive days. Note that the first day of participation is not always the first night in the hospital, although for most patients in both groups it was one of the first nights. Patients who were more recently admitted might have suffered more from the acute illness and had less time to adapt to the new environment, possibly leading to a so called “first night effect” (McCall and McCall, 2012). Finally, a considerable number of patients was asleep at the moment of potential inclusion. To optimize the inclusion rate, researchers visited the ward several times a day. Nevertheless, some patients were continuously asleep or too ill to participate, leading to a selection bias and possibly an overestimation or underestimation of the sleep quantity and quality found in this study (sleeping during the day may indicate insufficient sleep at night).

Although this study shows that the sleep quality and quantity in hospitalized patients suffering from an acute COVID-19 infection are insufficient, more research is needed to answer the question as to what degree these sleep disturbances are caused by the SARS-CoV-2 virus itself, or symptoms related to for example neurological or pulmonary manifestations of the infection. Furthermore, it would be interesting to re-conduct the study using polysomnography or actigraphy as soon as hygiene measures allow this. Finally, future studies should look into the effects of early detection of sleeping disorders during acute COVID-19 infection in preventing deterioration in at-risk patients and the positive effects of melatonin administration in enhancing the disrupted sleep-wake cycle in COVID-19 patients (Zambrelli et al., 2020).

## Conclusion

This study demonstrated that the mean sleep quality and quantity in both COVID-19 and non-COVID-19 patients was suboptimal (with a Total Sleep Time between 5 h and 5 h 30 min), but did not significantly differ between the groups. Nonetheless, with one in five COVID-19 patients reporting total sleep deprivation, patients in this group suffered five times more from extreme sleeping problems. Most patients did not suffer from insomnia 30 days before admission and the encountered problems can therefore be linked to the acute illness and/or hospitalization. Although great overlap exists, sleep disturbance in non-COVID-19 patients was mainly caused by environmental factors (noises of other patients, medical staff and medical devices) whereas the sleep of COVID-19 patients was relatively more often disturbed due to physical and psychological discomfort (dyspnea, concerns about their disease and anxiety). Results suggest that hospitalization with an acute COVID-19 infection may indeed be related to problems of sleep and alleviation should be sought in symptom relief, psychological support and noise reduction in the hospital environment.

## Data Availability Statement

The anonymized dataset will be made available on reasonable request after approval of the corresponding author.

## Ethics Statement

This study was part of a larger research project exploring the sleep of acutely admitted patients. The Executive Committee of the Medical Ethics Review Committee of VU University Medical Center (IRB00002991) decided that the Medical Research involving Human Subjects Act did not apply (reference no. 2019.246). The patients/participants provided their written informed consent to participate in this study.

## Author Contributions

EE, PN, and HM designed the study. EE, KV, BT, and NK were involved in recruiting participants and collecting the data. EE, KV, and PV were responsible for the statistical analyses. EE and KV wrote the first draft of the manuscript. All authors reviewed and approved the final version of the manuscript.

## Conflict of Interest

The authors declare that the research was conducted in the absence of any commercial or financial relationships that could be construed as a potential conflict of interest.

## Publisher’s Note

All claims expressed in this article are solely those of the authors and do not necessarily represent those of their affiliated organizations, or those of the publisher, the editors and the reviewers. Any product that may be evaluated in this article, or claim that may be made by its manufacturer, is not guaranteed or endorsed by the publisher.

## Abbreviations

COVID-19: Coronavirus Disease-19
SARS-CoV-2: Severe Acute Respiratory Syndrome Coronavirus-2
WHO: World Health Organization
CSD: Consensus Sleep Diary
TST: Total Sleep Time
SOL: Sleep Onset Latency
WASO: Wakefulness After Sleep Onset
NWAK: Number of Awakenings
TAFASA: Time Attempting to Sleep After Final Awakening
SE: Sleep Efficiency
PROMIS: Patient-Reported Outcomes Measurement Information System
ISI: Insomnia Severity Index
VAS: Visual Analog Scale
CFS: Clinical Frailty Scale
MEWS: Modified Early Warning Score
DOS: Delirium Observation Scale
CCI: Charlson Comorbidity Index
ICU: Intensive Care Unit
ED: Emergency Department
PCR: Polymerase Chain Reaction
SD: Standard Deviation
IQR: Inter-Quartile Range
SPSS: Statistical Package for Social Sciences
Min: Minutes
No: Number.
