# Supplementary material for: Hospitalized COVID-19 Patients Were Five Times More Likely to Suffer From Total Sleep Deprivation Compared to Non-COVID-19 Patients; an Observational Comparative Study
Source: Front Neurosci. 2021 Oct 5;15:680932. doi: 10.3389/fnins.2021.680932 (PMC8525610; doi:10.3389/fnins.2021.680932)
Supplement: Supplementary file 1 [file Data_Sheet_1.pdf]

Supplementary Material

**Supplementary Table 1.**	The use of potentially sleep affecting medication
**Supplementary Table 2.**	Patient status 30 days after inclusion
**Supplementary Table 3.**	Baseline characteristics and other relevant outcomes for COVID-19 patients that did not and those that did sleep.
**Supplementary Table 4.**	Sleep quantity for all five days (by means of the Consensus Sleep Diary)
**Supplementary Figure 1.**	The evolvement of Total Sleep Time for individual patients who completed five subsequent questionnaires
**Supplementary Figures 2a-c.**	Overview of all Sleep-Disturbing Factors
**Supplementary Table 5.**	Difference in sleep quality and quantity between COVID-19 patients with different complaints
**Supplementary Table 6.**	Spearman's correlations for Total Sleep Time and PROMIS total score
**Supplementary Figure 3.**	The correlation between the subjective well-being and quality of sleep

Supplementary Table 1. The use of potentially sleep affecting medication

Item	Day 1		Day 2		Day 3		Day 4		Day 5	
	COVID-19 (n=77)	Non-COVID-19 (n=50)	COVID-19 (n=55)	Non-COVID-19 (n=37)	COVID-19 (n=35)	Non-COVID-19 (n=25)	COVID-19 (n=21)	Non-COVID-19 (n=17)	COVID-19 (n=9)	Non-COVID-19 (n=12)
**Diuretic**	11 (14.3)	11 (20.4)	9 (16.4)	8 (21.6)	6 (17.1)	5 (20.0)	4 (19.0)	3 (17.6)	1 (11.1)	2 (16.7)
**Benzodiazepine**	11 (14.3)	9 (18.0)	11 (20.0)	4 (10.8)	9 (25.7)	4 (16.0)	5 (23.8)	3 (17.6)	0 (0.0)	3 (25.0)
**Melatonin**	1 (1.3)	0 (0.0)	0 (0.0)	0 (0.0)	0 (0.0)	0 (0.0)	0 (0.0)	0 (0.0)	0 (0.0)	0 (0.0)
**Valerian**	0 (0.0)	0 (0.0)	0 (0.0)	0 (0.0)	0 (0.0)	0 (0.0)	0 (0.0)	0 (0.0)	0 (0.0)	0 (0.0)
**Antidepressant**	2 (2.6)	3 (6.0)	1 (1.8)	2 (5.4)	0 (0.0)	1 (4.0)	0 (0.0)	0 (0.0)	0 (0.0)	0 (0.0)
**Antipsychotic**	1 (1.3)	0 (0.0)	1 (1.8)	0 (0.0)	1 (2.9)	0 (0.0)	0 (0.0)	0 (0.0)	0 (0.0)	0 (0.0)
**Antiepileptic**	4 (5.2)	3 (6.0)	3 (5.5)	3 (8.1)	1 (2.9)	0 (0.0)	1 (4.8)	0 (0.0)	0 (0.0)	0 (0.0)
**Antihistaminic**	5 (6.5)	0 (0.0)	3 (5.5)	1 (2.7)	0 (0.0)	1 (4.0)	0 (0.0)	1 (5.9)	1 (11.1)	1 (8.3)
**Opioid**	4 (5.1)	14 (28.0)	1 (1.8)	14 (37.8)	0 (0.0)	9 (36.0)	1 (4.8)	7 (41.2)	0 (0.0)	5 (41.7)
**Dexamethasone**	50 (64.9)	4 (8.0)	44 (80.0)	6 (16.2)	26 (74.3)	3 (12.0)	13 (61.9)	3 (17.6)	6 (66.7)	2 (16.7)
**(Methyl)-Prednisolone**	2 (2.6)	8 (16.0)	1 (1.8)	6 (16.2)	1 (2.9)	4 (16.0)	2 (9.6)	2 (11.8)	1 (11.1)	1 (8.3)

Data are presented as the number of patients (%).

Supplementary Table 2. Patient status 30 days after inclusion

Item	COVID-19 (n=79)	Non-COVID-19 (n=49)	P-value
**Patients status**[a]			
Discharged	73 (94.9)	48 (98.0)	
Still Hospitalized	0 (0.0)	0 (0.0)	0.648
Deceased	4 (5.1)	1 (2.0)	
**Length of Hospital stay**, days Median [IQR]	5.5 [4.0-9.25]	5.0 [4.0-10.0]	0.907
**Discharged to**			
Own living environment	54 (69.2)	45 (91.8)	
Rest home/nursing home	13 (16.6)	2 (4.1)	
Rehabilitation home	6 (7.7)	2 (4.1)	0.076
Deceased during hospitalization	3 (3.8)	0 (0.0)	
Transfer ward	2 (2.6)	0 (0.0)	
**Unplanned ICU admission**	10 (12.8)	2 (4.1)	0.127
**Delirious**	0 (0.0)	3 (6.1)	0.055
**Readmitted within 30 days after inclusion**	6 (7.6)	9 (18.0)	0.077

Abbreviations: IQR, interquartile range; ICU, Intensive Care Unit.
Data are presented as the number of patients (%), unless stated otherwise.
[a]For this question there were two missing values in the COVID-19 group, and one missing value in the non-COVID-19 group.

Supplementary Table 3. Baseline characteristics and outcomes for COVID-19 patients that did and did not sleep.

Characteristic	0 hours of sleep (n=13)	≥ 1 hours of sleep (n=66)	P-value
**Gender**, male, n (%)	9 (69.2)	38 (57.6)	0.544
**Age**, median [IQR]	73.0 [63.0-83.0]	62.0 [53.8-72.0]	0.010
**Other patients in the room**, median [IQR]	2.0 [0.5-2.5]	2.0 [1.0-3.0]	0.381
**Other patients in the room**, n (%)			
0	3 (23.1)	10 (15.4)	
1	1 (7.7)	8 (12.3)	0.633
2	6 (46.2)	22 (33.8)	
3	3 (23.1)	25 (38.5)	
**Length of Stay,** days, median [IQR]	5.0 [3.5-9.0]	6.0 [4.0-10.0]	0.458
**No. of nights hospitalized at first night of participation**, median [IQR]	1.5 [1.0-2.3]	2.0 [2.0-3.0]	0.089
**Location of patient before inclusion,** n (%)			
Home / Nursing Home / Rehabilitation Centre	10 (76.9)	41 (62.1)	
Intensive Care Unit	1 (7.7)	7 (10.6)	0.806
Other ward / hospital (not ED)	2 (15.4)	17 (25.8)	
Missing	0 (0.0)	1 (1.5)	
**History of Insomnia**[a]**,** n (%)			
Absence of insomnia	12 (92.3)	40 (63.5)	
Sub-threshold insomnia	1 (7.7)	11 (17.5)	0.287
Moderate insomnia	0 (0.0)	9 (14.3)	
Severe insomnia	0 (0.0)	3 (4.8)	
**Clinical Frailty Scale**[b], median [IQR]	4 [3-5]	3 [2-4]	0.037
**Charlson Comorbidity Index**[c]**,** median [IQR]	3.0 [2.5-4.0]	2.0 [1.0-4.8]	0.445
**Modified Early Warning Score**[d]**,** median [IQR]	1.0 [0.0-1.0]	0.5 [0.0-1.0]	0.458
**No. of patients that received oxygen therapy at day 1,** n (%)	10 (76.9)	48 (75.0)	1.000
**Liters oxygen/minute,** median [IQR]	3.8 [2.0-17.8]	3.0 [2.0-5.0]	0.335
**Patients' status 30 days after inclusion,** n (%)			
Discharged	11 (84.6)	63 (95.5)	
Deceased	2 (15.4)	2 (3.0)	0.127
Missing	0 (0.0)	1 (1.5)	
**Neurological complaints**, n (%)			
0	4 (30.8)	13 (19.7)	0.461
≥ 1	9 (69.2)	53 (80.3)	
**Pulmonary complaints,** n (%)			
0	2 (15.4)	9 (13.6)	0.100
≥ 1	11 (84.6)	57 (86.4)	
**Visual Analogue Scales**[e], median [IQR]			
Depression	5.0 [1.0-8.0]	4.0 [1.0-7.0]	0.952
Anxiety	4.0 [1.0-8.8]	4.0 [1.0-7.0]	0.219
Pain	1.0 [1.0-2.5]	1.0 [1.0-4.0]	0.455
Dyspnea in rest	2.0 [1.0-7.0]	3.0 [1.0-6.0]	0.971
Dyspnea when walking to the toilet	5.0 [1.0-9.5]	6.0 [2.0-9.0]	0.471

Abbreviations: IQR, interquartile range; ED, Emergency Department.

[a]Measured by means of the Insomnia Severity Index (ISI), resulting in a total score ranging from 0 to 28. Absence of insomnia (0-7), sub-treshold insomnia (8-14), moderate insomnia (15-21) and severe insomnia (22-28).

[b]The Clinical Frailty Scale (CFS) is used to rate patients' pre-admission frailty. Scores range from 1 (very fit) to 9 (terminally ill).

[c]The Charlson Comorbidity Index (CCI) results in a total score ranging from 0 to 24. No comorbidity (0), mild comorbidity

(1-2), moderate comorbidity (3-4), severe comorbidity (≥5).
[d]The Modified Early Warning Score (MEWS) is mainly based on vital parameters (i.e. systolic blood pressure, heart rate, respiratory rate, temperature, AVPU score) and used for identification of patients at risk of deterioration. Each parameter is scored from 0 to 3, resulting in a total score ranging from 0 (best) to 14 (worst).
[e]All VAS scores result in a total score ranging from 0-10, whereat 0 represents not being depressed, no anxiety, no pain and no shortness of breath. The maximum score of 10 stands for feeling depressed, being very anxious, feeling the worst possible pain and being maximally short of breath.

Supplementary Table 4. Sleep quantity for all five days (by means of the Consensus Sleep Diary)

	Day 1			Day 2			Day 3			Day 4			Day 5		
	**COVID-19 (n=79)**	**Non-COVID-19 (n=50)**	**Median Difference (p-value)**	**COVID-19 (n=55)**	**Non-COVID-19 (n=36)**	**Median Difference (p-value)**	**COVID-19 (n=35)**	**Non-COVID-19 (n=25)**	**Median Difference (p-value)**	**COVID-19 (n=21)**	**Non-COVID-19 (n=17)**	**Median Difference (p-value)**	**COVID-19 (n=10)**	**Non-COVID-19 (n=12)**	**Median Difference (p-value)**
**Closing Eyes to Sleep Time**, h:m	23:00 [22:00 – 00:00][c]	23:15 [22:45-23:52][a]	-0:15 (0.083)	23:00 [22:00-24:00]	23:00 [22:30-23:55]	0:00 (0.140)	22:52 [22:00-00:11][a]	23:30 [22:15-00:00]	-0:38 (0.638)	23:00 [22:22-00:07]	23:00 [22:00-23:30]	0:00 (0.281)	23:00 [22:30-23:45][a]	22:45 [22:15-23:52]	00:15 (0.828)
**Sleep Onset Latency[1]**, min.	00:15 [00:10-01:00][j]	00:30 [00:10-00:53][e]	-00:15 (0.611)	00:25 [00:06-01:00][a]	00:15 [00:05-00:30][a]	00:10 (0.200)	00:15 [00:10-00:35]	00:15 [00:06-01:00][a]	00:00 (0.770)	00:10 [00:05-00:30][d]	00:15 [00:10-00:25]	-00:05 (0.326)	00:08 [00:05-00:30][b]	00:13 [00:06-00:15]	-00:05 (0.553)
**Number of Awakenings** No.	2.0 [1.0-4.0]	3.0 [1.0-5.0]	-1 (0.477)	2.0 [1.0-3.0][f]	2.0 [1.0-3.0]	0.0 (0.935)	1.5 [0.25-3.0][c]	2.0 [1.0-3.0][a]	-0.5 (0.335)	1.5 [1.0-3.0][c]	2.0 [0.5-2.5]	-0.5 (0.838)	2.0 [1.25-5.75][b]	2.0 [1.0-2.0]	0.0 (0.212)
**Wake After Sleep Onset[2]**, min.	00:30 [00:10-01:30]	00:40 [00:10-01:23][b]	-00:10 (0.900)	00:30 [00:05-01:00][h]	00:43 [00:05-01:30][d]	-00:13 (0.301)	00:20 [00:00-00:45][d]	00:26 [00:07-00:56][a]	-00:05 (0.484)	00:15 [00:10 - 00:55][e]	00:30 [00:02-01:00]	-00:15 (0.514)	00:40 [00:10-01:18][b]	00:24 [00:11-00:41]	00:16 (0.615)
**Final Wake Time,** h:m	6:20 [5:30-7:00][e]	6:20 [6:00-7:00][a]	0:00 (0.919)	6:00 [5:30-7:00]	6:00 [5:37-6:30]	0:00 (0.949)	6:00 [5:30-6:45][b]	6:00 [5:30-6:30][2]	0:00 (0.769)	5:45 [5:30-6:45][d]	6:00 [5:55-7:22]	-0:15 (0.163)	6:30 [5:07-8:11][b]	6:30 [6:03-7:22]	0:00 (0.786)
**Sleep Episode[3]**, hh:mm	07:15 [06:00-08:30]	07:13 [06:00-08:00]	00:02 (0.985)	07:00 [06:08-08:28][b]	07:00 [05:30-08:00][a]	00:00 (0.405)	07:30 [05:55-08:03][b]	06:30 [06:00-07:15][c]	01:00 (0.599)	07:15 [05:45-08:45][d]	07:05 [06:30-08:58]	00:10 (0.448)	07:15 [06:08-09:34][b]	07:30 [06:49-08:04]	-00:15 (0.728)
**Total Sleep Time[4]**, hh:mm	05:43 [02:24-07:40][i]	06:00 [04:15-06:50][e]	-00:18 (0.618)	05:55 [03:45-07:40][h]	05:45 [04:15-07:00[e]	00:10 (0.898)	05:35 [03:45-07:23][b]	05:49 [04:36-06:31][c]	-00:14 (0.757)	06:05 [02:25-07:40][d]	06:28 [05:19-07:43]	-00:23 (0.460)	05:25 [01:08-07:40]	06:36 [06:10-07:24]	-00:71 (0.222)
**Sleep Efficiency[5]**, %	82.64 [31.41-93.01][g]	82.35 [64.58-94.91][e]	0.29 (0.283)	84.95 [60.00-93.59][h]	87.04 [57.58-95.45][e]	-2.1 (0.721)	89.74 [65.39-95.84][b]	88.86 [73.48-96.68][c]	0.88 (0.938)	84.81 [55.21-95.79][d]	86.48 [77.75-93.16][a]	-1.67 (0.943)	85.00 [16.07-91.60]	93.09 [78.91-94.58]	-8.09 (0.099)
**Time Attempting to Sleep after Final Awakening**, min.	00:30 [00:00-01:30][h]	00:00 [00:00-00:49]	00:30 (0.111)	00:15 [00:00-01:30][f]	00:05 [00:00-01:00][a]	00:10 (0.120)	X	X	X	X	X	X	X	X	X
**Daytime Sleep**, hh:mm	00:00 [00:00 - 01:37]	00:00 [00:00-01:00]	00:00 (0.104)	00:00 [00:00 01:00]	00:00 [00:00 - 01:00]	00:00 (0.431)	X	X	X	X	X	X	X	X	X

Abbreviations: IQR, interquartile range; h:m, hours:minutes;
Differences are calculated by subtracting non-COVID-19 scores from COVID-19 scores.
Data are summarized as median [IQR].

[1]Time it took to fall asleep after closing eyes to sleep.
[2]Time spent awake after onset of sleep
[3]Time interval from "Closing eyes to sleep" until "final awakening"
[4]The time spent asleep within the sleep episode
[5]The percentage of sleep time within the sleep episode (TST/SE (*100))

Some questions contain missing values; [a]one missing value, [b]two missing values, [c]three missing values, [d]four missing values, [e]five missing values, [f]six missing values, [g]seven missing values, [h]eight missing values, [i]nine missing values, [j]ten missing values.

Supplementary Figure 1. The evolvement of Total Sleep Time for individual patients who completed five subsequent questionnaires

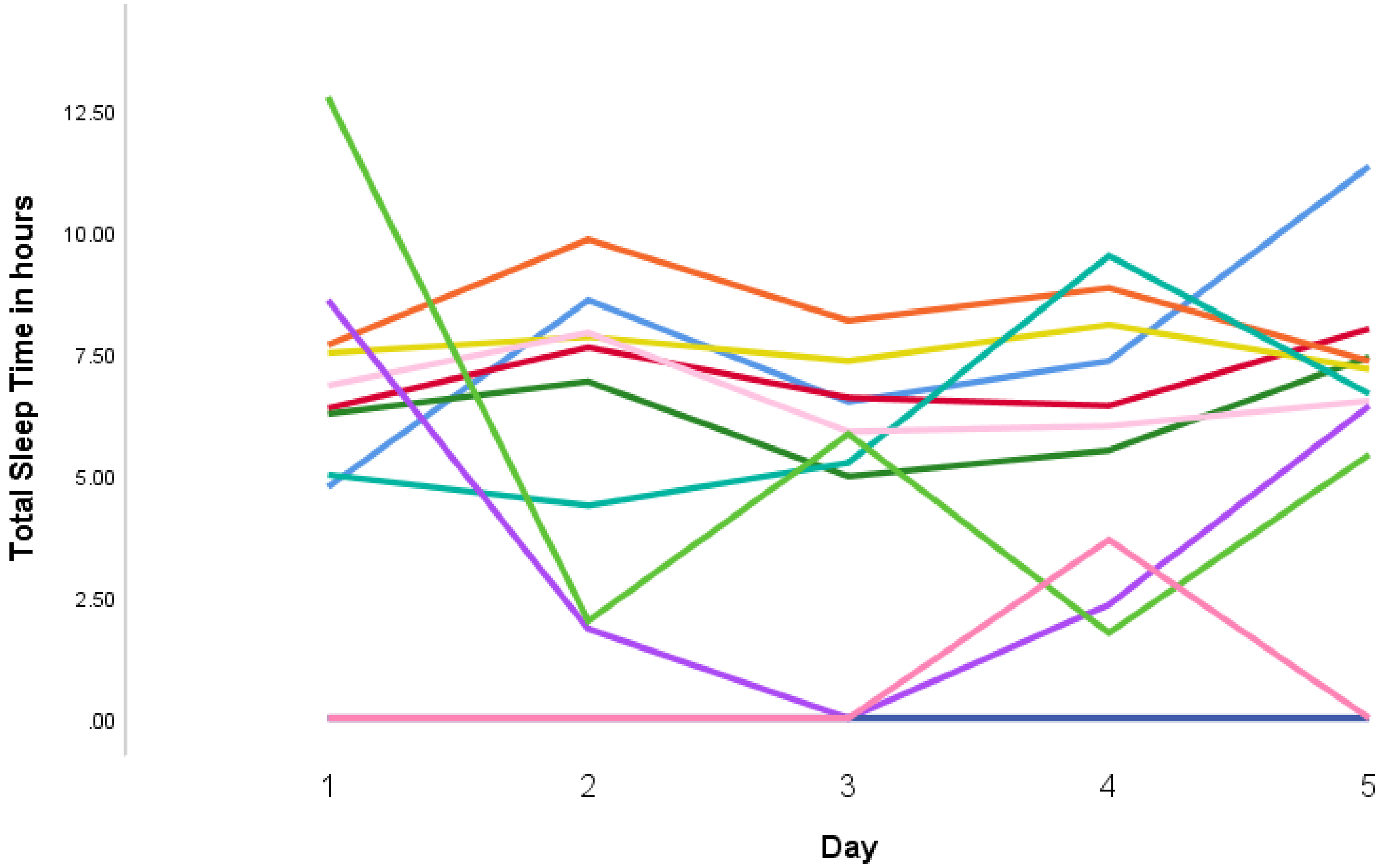

Supplementary Figures 2a-c. Overview of all Sleep-Disturbing factors

Figure 2a

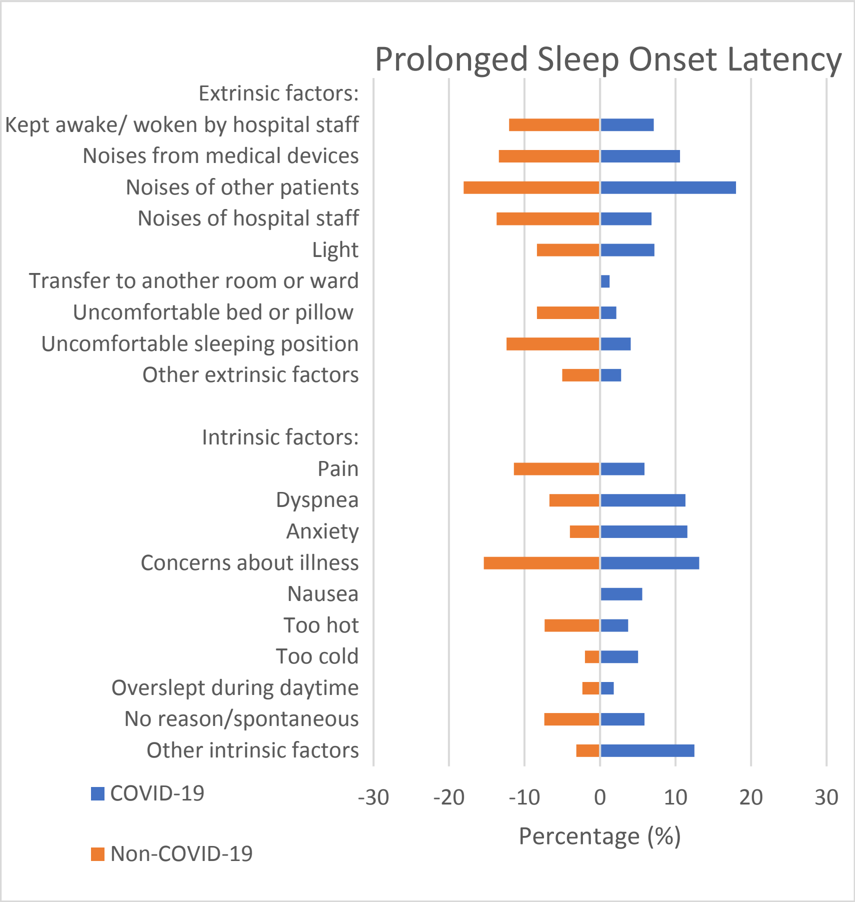

Figure 2b

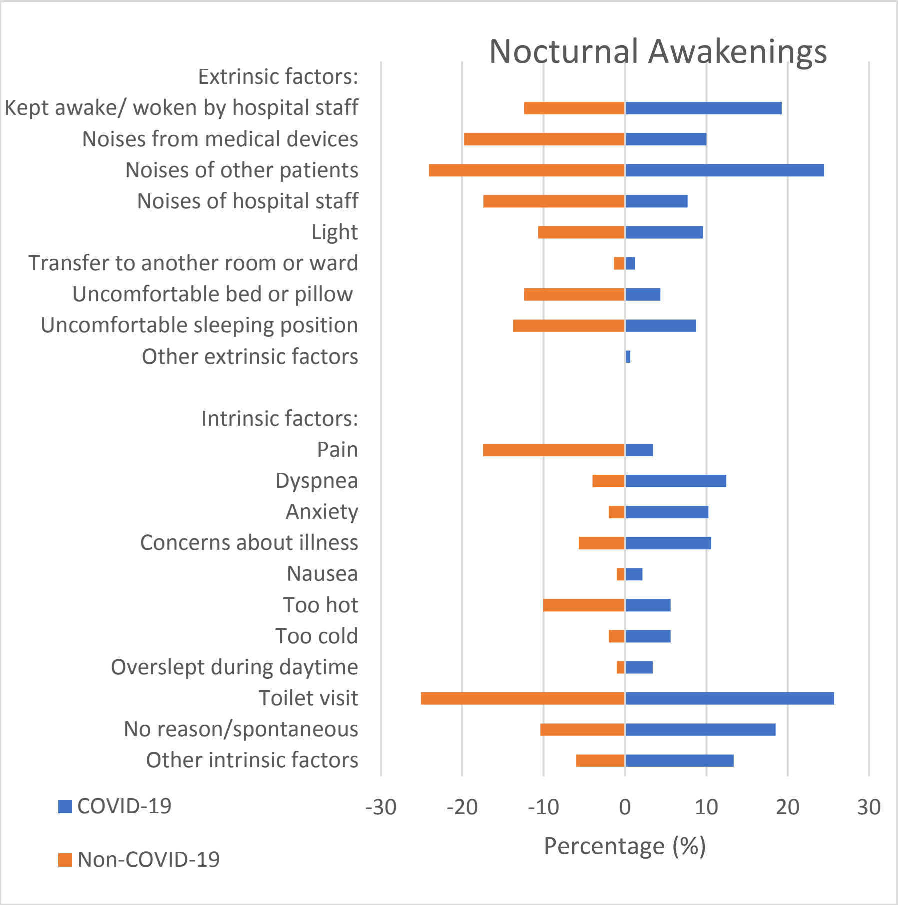

Figure 2c.

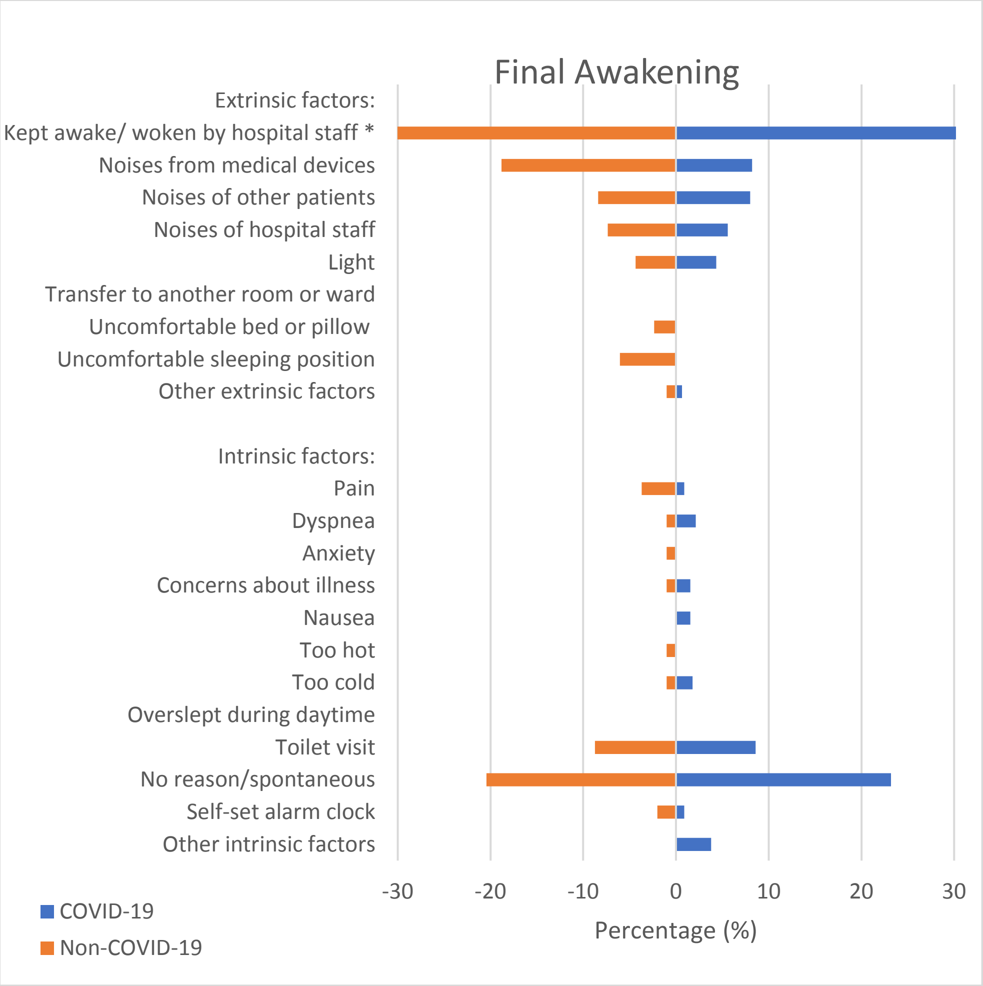

Data shown are the patient-reported disturbing factors reported by patients of night one and two combined. Patients were allowed to assign multiple sleep disturbing factors to why they experienced a prolonged sleep onset latency and/ or nocturnal awakenings. Only one answer could be assigned to what caused their final awakening. Percentages are the proportion of all patients in that sub-group (COVID-19 versus patients without COVID-19) that filled in the questionnaire for that day. “Other extrinsic factors” were e.g. medication, discomfort due to medical devices (i.e. oxygen mask, drips). “Other intrinsic factors” were e.g. general complaints due to COVID-19 infection, symptoms (i.e. coughing, headaches, dizziness, gastro-intestinal reflux, sweating).
*The actual percentage is 55.1% in the COVID-19 group and 55.4% in the non-COVID-19 group.

Supplementary Table 5. Difference in sleep quality and quantity between COVID-19 patients with different complaints

	Day 1						Day 2					
	**COVID-19 (n=79)**		**Difference (p-value)**	**Non-COVID-19 (n=50)**		**Difference (p-value)**	**COVID-19 (n=55)**		**Difference (p-value)**	**Non-COVID-19 (n=36)**		**Difference (p-value)**
	**No complaints**	**≥ 1 complaint**		**No complaints**	**≥ 1 complaint**		**No complaints**	**≥ 1 complaint**		**No complaints**	**≥ 1 complaint**	
**Pulmonary complaints[1]**												
Total Sleep Time[a], Mean (SD)	05:16 (03:43)	05:04 (00:47)	00:12 (0.765)	05:55 (01:39)	05:25 (00:47)	00:30 (0.711)	06:14 (01:52)	05:08 (00:57)	01:06 (0.541)	05:40 (01:23)	05:15 (01:13)	00:40 (0.598)
PROMIS Total Score[b] Median [IQR]	21.0 [17.0-31.0]	26.0 [17.0-36.0]	-5.0 (0.336)	21.0 [15.3-35.0]	24.0 [18.0-28.0]	-3.0 (0.716)	15.0 [12.0-26.0]	25.5 [16.8-34.3]	-10.5 (0.031)	16.0 [14.0-27.0]	22.0 [14.8-29.8]	-6.0 (0.493)
**Neurological complaints[2]**												
Total Sleep Time[a] Mean (SD)	04:22 (01:59)	05:17 (00:52)	-00:55 (0.389)	05:57 (00:54)	04:12 (01:03)	01:45 (0.217)	05:32 (01:50)	05:18 (00:58)	00:14 (0.651)	05:45 (01:12)	05:03 (01:22)	00:42 (0.395)
PROMIS Total Score[b] Median [IQR]	21.0 [17.0-35.5]	26.0 [19.0-35.5]	-5.0 (0.396)	22.0 [13.5-27.3]	25.0 [19.3-32.0]	-3.0 (0.180)	17.0 [13.0-29.0]	26.0 [16.8-34.5]	-9.0 (0.058)	15.0 [14.0-24.0]	24.5 [18.3-33.8]	-9.5 (0.037)
**Abdominal complaints[3]**												
Total Sleep Time[a] Mean (SD)	05:05 (00:54)	05:08 (01:51)	00:03 (0.742)	05:34 (00:45)	05:14 (01:59)	00:20 (0.618)	05:43 (00:55)	03:51 (02:04)	01:52 (0.068)	05:20 (00:51)	05:49 (04:40)	-00:29 (0.468)
PROMIS Total Score[b] Median [IQR]	26.0 [17.3-34.8]	28.5 [16.5-37.0]	-2.5 (0.934)	23.0 [17.3-28.0]	27.5 [20.3-31.8]	-4.5 (0.463)	21.0 [14.0-33.0]	26.0 [15.8-38.5]	-5.0 (0.413)	22.0 [14.8-30.3]	19.0 [10.0-22.0]	3.0 (0.194)
**Muscle / joint complaints[4]**												
Total Sleep Time[a] Mean (SD)	04:57 (00:57)	05:21 (01:29)	-00:24 (0.213)	05:19 (00:54)	06:07 (00:58)	-00:48 (0.810)	05:11 (00:52)	05:54 (02:16)	-00:43 (0.267)	05:16 (00:57)	06:12 (03:05)	00:56 (0.519)
PROMIS Total Score[b] Median [IQR]	24.0 [17.0-34.0]	27.0 [22.0-38.0]	-3.0 (0.165)	23.0 [18.0-21.8]	23.5 [18.8-28.0]	-0.5 (0.665)	22.5 [15.3-32.5]	23.0 [14.0-36.0]	-0.5 (0.702)	21.0 [15.0-27.0]	23.5 [10.0-32.0]	-2.5 (0.890)

Abbreviations: PROMIS, Patient-Reported Outcomes Measurement Information System; SD, standard deviation; IQR, interquartile range

Differences indicate no complaints minus ≥ 1 complaint.

[a]TST is presented in hours and minutes,

[b]PROMIS total score ranges from 8-40, a higher score representing more sleep disturbance.

[1]Pulmonary complaints contain coughing, dyspnea, mucus. [2]Neurological complaints contain headache, fatigue, memory loss and fever. [3]Abdominal complaints contain abdominal pain, vomiting, diarrhea. [4]Muscle / joint complaints contain muscle pain and joint pain.

Supplementary Table 6. Spearman's correlations for Total Sleep Time and PROMIS total score

Item	COVID-19 (n=79)		Non-COVID-19 (n=49)	
	r	p-value	r	p-value
**Total Sleep Time**				
Depression	-0.128	0.290	0.091	0.551
Anxiety	-0.161	0.185	-0.121	0.429
Pain	0.659	0.064	-0.089	0.561
Dyspnea in rest	0.097	0.433	-0.014	0.929
Dyspnea when walking	0.230	0.151	-0.019	0.912
PROMIS	-0.542	0.000	-0.711	0.000
Length of Stay	0.303	0.011	0.146	0.344
Unplanned ICU admission	0.147	0.227	-0.030	0.846
30-day mortality	0.023	0.852	-0.134	0.382
**PROMIS**				
Depression	0.303	0.007	0.128	0.376
Anxiety	0.273	0.017	0.024	0.866
Pain	-0.052	0.659	0.324	0.022
Dyspnea in rest	0.049	0.676	0.228	0.111
Dyspnea when walking	0.155	0.230	0.091	0.568
Length of Stay	-0.113	0.328	-0.255	0.077
Unplanned ICU admission	-0.005	0.962	0.018	0.901
30-day mortality	-0.001	0.9931	0.183	0.202

Abbreviations: PROMIS, Patient-Reported Outcomes Measurement Information System; ICU, Intensive Care Unit.

Supplementary Figure 3. The correlation between the subjective well-being and quality of sleep

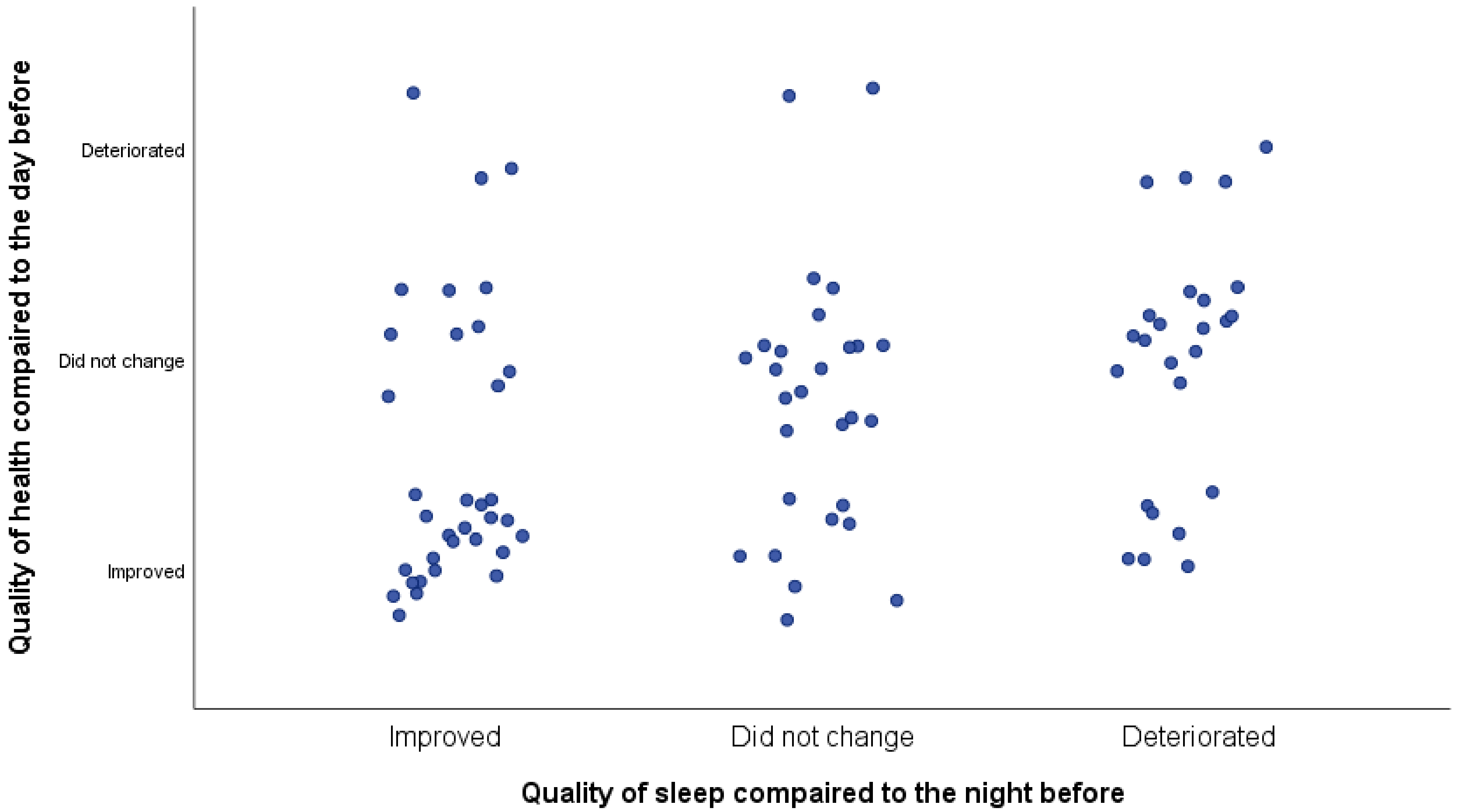

Patient reported physical well-being (compared to the day before) correlated with a patient reported experienced sleep quality (compared to the night before). Spearman's Rho: (r 0.300 *p=0.005*)
